# Local Increase of Arginase Activity in Lesions of Patients with Cutaneous Leishmaniasis in Ethiopia

**DOI:** 10.1371/journal.pntd.0001684

**Published:** 2012-06-12

**Authors:** Tamrat Abebe, Asrat Hailu, Mihretu Woldeyes, Woinshet Mekonen, Kassahun Bilcha, Thomas Cloke, Lionel Fry, Nafisa-Katrin Seich al Basatena, Karina Corware, Manuel Modolell, Markus Munder, Fabienne Tacchini-Cottier, Ingrid Müller, Pascale Kropf

**Affiliations:** 1 Department of Microbiology, Immunology and Parasitology, Addis Ababa University, Addis Ababa, Ethiopia; 2 Department of Biochemistry, WHO Immunology Research and Training Center, University of Lausanne, Lausanne, Switzerland; 3 Department of Dermatology and Venerology, Addis Ababa University, Addis Ababa, Ethiopia; 4 Department of Dermatovenerology, Gondar University, Gondar, Ethiopia; 5 Section of Immunology, Department of Medicine, Imperial College London, London, United Kingdom; 6 Department of Medicine, Imperial College London, London, United Kingdom; 7 Department of Cellular Immunology, Max-Planck-Institute for Immunobiology and Epigenetics, Freiburg, Germany; 8 Third Department of Medicine (Hematology, Oncology, and Pneumology), University Medical Center Mainz, Mainz, Germany; 9 Infection and Immunology Department, London School of Hygiene and Tropical Medicine, London, United Kingdom; René Rachou Research Center, Brazil

## Abstract

**Background:**

Cutaneous leishmaniasis is a vector-borne disease that is in Ethiopia mainly caused by the parasite *Leishmania aethiopica*. This neglected tropical disease is common in rural areas and causes serious morbidity. Persistent nonhealing cutaneous leishmaniasis has been associated with poor T cell mediated responses; however, the underlying mechanisms are not well understood.

**Methodology/Principal Findings:**

We have recently shown in an experimental model of cutaneous leishmaniasis that arginase-induced L-arginine metabolism suppresses antigen-specific T cell responses at the site of pathology, but not in the periphery. To test whether these results translate to human disease, we recruited patients presenting with localized lesions of cutaneous leishmaniasis and assessed the levels of arginase activity in cells isolated from peripheral blood and from skin biopsies. Arginase activity was similar in peripheral blood mononuclear cells (PBMCs) from patients and healthy controls. In sharp contrast, arginase activity was significantly increased in lesion biopsies of patients with localized cutaneous leishmaniasis as compared with controls. Furthermore, we found that the expression levels of CD3ζ, CD4 and CD8 molecules were considerably lower at the site of pathology as compared to those observed in paired PBMCs.

**Conclusion:**

Our results suggest that increased arginase in lesions of patients with cutaneous leishmaniasis might play a role in the pathogenesis of the disease by impairing T cell effector functions.

## Author Summary

The leishmaniases are a complex of diseases caused by *Leishmania* parasites. Currently, the diseases affect an estimated 12 million people in 88 countries, and approximately 350 million more people are at risk. The leishmaniases belong to the most neglected tropical diseases, affecting the poorest populations, for whom access to diagnosis and effective treatment are often not available. *Leishmania* parasites infect cells of the immune system called macrophages, which have the capacity to eliminate the intracellular parasites when they receive the appropriate signals from other cells of the immune system. In nonhealing persistent leishmaniasis, lymphocytes are unable to transmit the signals to macrophages required to kill the intracellular parasites. The local upregulation of the enzyme arginase has been shown to impair lymphocyte effector functions at the site of pathology. In this study, we tested the activity of this enzyme in skin lesions of patients presenting with localized cutaneous leishmaniasis. Our results show that arginase is highly upregulated in these lesions. This increase in arginase activity coincides with lower expression of a signalling molecule in lymphocytes, which is essential for efficient activation of these cells. These results suggest that increased arginase expression in the localized cutaneous lesions might contribute to persistent disease in patients presenting with cutaneous leishmaniasis.

## Introduction

The leishmaniases are a complex of vector-borne diseases caused by the parasite *Leishmania*. They are neglected tropical diseases, that affect the poorest population and cause major morbidity and mortality, estimated to 2.4 million disability-adjusted life-years [1]. Currently, these diseases affect an estimated 12 million people in 88 countries, and approximately 350 million people are at risk [2]. Leishmaniases can present with a wide range of clinical syndromes that may be cutaneous or visceral: cutaneous leishmaniasis (CL) is manifested as localized (LCL), mucocutaneous (MCL) or mucosal (ML) and diffuse (DCL) disease [3]. Visceral leishmaniasis (VL), the most severe form of leishmaniasis, is a systemic disease, in which the mortality rate can be as high as 100% if left untreated. Adequate treatment results in an overall cure rate of >90% [3].

Leishmaniasis is one of the most important vector-borne diseases in Ethiopia, where it is mainly prevalent in the highlands. However, there is still only very limited information from epidemiological studies about the number of VL and CL cases. According to the Ethiopian National Guidelines for the Diagnosis and Treatment of Leishmaniasis, Ethiopia has the second largest number of VL cases in sub-Saharan Africa with an estimated 4500 to 5000 new cases every year. VL is associated with high mortality and morbidity, and is worsened by poor nutrition, isolated location of VL endemic areas and co-infections with HIV [4]. Similarly, there is limited data about the frequency and distribution of CL in Ethiopia [5], [6], [7], [8], [9], [10], [11]. CL in Ethiopia is mainly caused by *Leishmania* (*L.*) *aethiopica*, and rarely by *L. tropica* or *L. major* [12] and can manifest as LCL, with localized cutaneous nodular lesions, that can ulcerate and heal, leaving depressive scars (LCL); DCL, which is characterized by disseminated nodular lesions; and MCL, with lesions spreading into the nasal and/or oral mucosa [13]. LCL usually heals spontaneously within 1 year [7], however, persistent LCL as well as MCL and DCL require treatment; relapses are frequent after treatment in DCL and MCL [3], [12].

One experimental model of cutaneous leishmaniasis caused by *L. major* has been extensively studied: in this model, infection of BALB/c mice induces progressive nonhealing lesions; this inability to control infection has been associated with a polarized T helper (Th) 2 response. In contrast, C57BL/6 or CBA mice can efficiently control parasite replication and become immune to secondary challenge, this has been ascribed to a Th1 response [14], [15], [16]. In sharp contrast, infections of different strains of mice and other rodents with *L. aethiopica* does not lead to obvious clinical symptoms, even though parasites can be isolated from *L. aethiopica* infected BALB/c mice [17], [18], [19]. The exceptions are the Syrian and the CBC hamsters, which can be successfully infected into the nose, and produce lesions similar to those observed in DCL patients [19]. There is also very limited information on the immune response in *L. aethiopica* infected patients. It has been shown that LCL, but not DCL patients will respond to leishmanin skin test [20]. Furthermore, whereas mononuclear cells from LCL patients can proliferate and express cytokines *in vitro* in response to antigenic restimulation, those from DCL patients have an impaired capacity to become activated [20], [21], [22]. While the mechanisms responsible for this hyporesponsiveness are not yet clarified, it has been suggested that lower levels of IFN-γ and increased expression of IL-10 might contribute to immunosuppression in DCL patients [21]. CD8^+^ T cells and NK cells may also play a protective role [23].

The catabolism of L-arginine by arginase is emerging as a critical mechanism of immune regulation [24], [25], [26]. Arginase, which is typically considered to be an enzyme of the urea cycle in the liver, hydrolyzes L-arginine to urea and ornithine, which is further metabolized into polyamines. Arginase can be upregulated by cytokines such as IL-4 and IL-13, which can synergize with IL-10 and IL-21, as well as by inflammatory stimuli (summarized in [26]).

Upregulation of arginase in myeloid cells results in increased uptake of extracellular L-arginine, thus reducing L-arginine levels in the microenvironment. Since T cells unconditionally require L-arginine for efficient activation, decrease in L-arginine results in impaired T cell responses [24], [25], [26]. The downregulation of T cell responses by arginase-induced L-arginine depletion has been studied in several cancer models [25], in corneal transplantation [27] and pregnancy [28] and increased arginase activities have been associated with a variety of infectious diseases such as schistosomiasis [29], trypanosomiasis [30], tuberculosis [31], leishmaniasis [32], [33], hepatitis B [34] and HIV [35].

We have recently shown in an experimental model of cutaneous leishmaniasis that high arginase activity is a hallmark of nonhealing disease [32] and that this increased arginase contributes to persistent nonhealing leishmaniasis by causing local suppression of T cell responses [33].

To determine whether our experimental data translate to human disease, we tested whether enhanced arginase activity is present in biopsies of LCL patients and whether this coincides with T cell suppression.

## Methods

### Subjects and sample collection

The study was approved by the Ethiopian National Research Ethics Review Committee (NRERC, reference 310/18/03), by Addis Ababa University Medical Faculty Institutional Review Board (IRB, reference 023/2009) and by the Joint UCL/UCLH Committees on the Ethics of Human Research (Committee Alpha, reference 09/H0715/93). For this study, a cohort of 15 patients with localized cutaneous leishmaniasis was recruited from the Leishmaniasis Research and Diagnostic Laboratory, Addis Ababa University, Ethiopia. Ten healthy controls were recruited among the staff of the hospital; they had careful physical examinations and showed no cutaneous lesions and had no prior history of cutaneous leishmaniasis. Informed written consent was obtained from each patient and control and all data analyzed were anonymized. 8–20 ml of blood in EDTA tubes and 1 or 2 biopsies (3 or 4 mm) were collected from each patient from the edge of the active lesion before the treatment started; or from intact skin on one forearm from the healthy controls. Patients positive for HIV were excluded from the study. Both the biopsies and blood were processed immediately after harvesting. Peripheral blood mononuclear cells (PBMCs) were isolated by density gradient centrifugation on Histopaque®-1077 (Sigma). Cells were washed in phosphate buffered saline (PBS) and were immediately used for flow cytometry; PBMCs used for arginase and protein determination were immediately resuspended in lysis buffer (0.1% Triton X-100, 25 mM Tris-HCl and 10 mM MnCl_2_, Sigma) and then frozen at −20°C until further use. Biopsies were collected in PBS and homogenized in PBS for flow cytometry or in lysis buffer and frozen until arginase and protein assays were performed.

### Determination of arginase activity

The enzymatic activity of arginase was measured as previously described [35]. Briefly, cell lysates were activated by heating for 10 min at 56°C. L-arginine hydrolysis was conducted by incubating the activated lysates with 50 µL 0.5 M L-arginine (pH 9.7) at 37°C for 60 min. The reaction was stopped with 400 µL H_2_SO_4_ (96%)/H_3_PO_4_(85%)/H_2_O (1∶3∶7, v/v/v). Twenty µL α-isonitrosopropiophenone (ISPF, dissolved in 100% ethanol, Sigma) was added and incubated for 45 min at 100°C, followed by 30 min at 4°C. The optical density (OD) was measured at 550 nm. One unit of enzyme activity is defined as the amount of enzyme that catalyzes the formation of 1 µmol of urea per min.

### Protein determination

To determine the protein concentration of each PBMC sample, serial dilutions of each PBMC sample were made in PBS (Sigma). BCA Protein Assay Reagent (Pierce) was added to each PBMC dilution following supplier's recommendations. A bovine serum albumin (BSA) standard (Pierce) was serially diluted using PBS. Following 30 min incubation at 37°C, the optical density (OD) was measured at 570 nm.

### Flow cytometry

Antibodies used were as follows: anti-CD4 (clone 13B8.2, Beckman Coulter), anti-CD8 (clone RPA-T8, BD Biosciences), anti-CD3ζ (Santa Cruz: clone 6B10.2), anti-CD14 (BD Pharmingen: cloneM5E2), anti-CD15 (Clone H198, BD Pharmingen); anti-arginase I (HyCult Biotechnology: clone 6G3) and the isotype control (BD Pharmingen: clone MOPC21) were coupled with Alexa FluorR 488 (Molecular Probes). Cells were washed with PBS, the fixation step was performed with 2% formaldehyde in PBS and the permeabilisation step with 0.5% saponin in PBS.

The determination of intracellular arginase was performed as described in [35]. The percentages for the isotype controls were <1.5%. Acquisition was performed using a FACSCalibur (BD Biosciences) and data were analyzed using Summit v4.3 software.

### Statistical analyzes

Data were evaluated for statistical differences using a two-tailed Mann-Whitney test, Wilcoxon pair test or Spearman's rank test when appropriate (GraphPad Prism 5) and differences were considered statistically significant at *p*<0.05.

## Results

### Clinical data

Fifteen patients with lesions of LCL that were typical in their history and appearance were recruited for this study. All patients lived or had travelled in regions of Ethiopia endemic for CL caused by *L. aethiopica*; however, the infecting parasites were not typed. The diagnosis was confirmed by demonstration of amastigotes in skin scraping and growth of promastigotes in NNN medium. Out of the 15 patients recruited in this study, 7 were female and 8 were male, with a median age of 19±2.4 (Table 1). The large majority of the patients presented with nodular lesions (13 patients), 1 patient with an ulcerated lesion and 1 patient with ulcerated and nodular mixed lesions. Ten patients had 1 lesion and 5 patients had 2 lesions. The majority of the lesions (12) were found on the face (forehead, ear, cheek, lip, nose), 3 on the forearm, 1 on the neck and 1 on a finger. The duration of their illness ranged from 4 to 48 months (median ± SEM: 12 months±3.6): 7 patients had lesions for <12 months and 8 patients had lesions for >12 months (Table 1).

**Table 1:** Clinical data.

Patient	Sex	Age	Lesion type	Lesion size (mm)	# lesions	Lesion site	Duration of illness (months)
1	F	17	ulcerated	nd	1	Forearm	12
2	M	17	nodular	24×23	1	Nose	3
3	F	20	nodular	35×20/22×22	2	Nose/forearm	36
4	F	13	nodular	40×50/10×10	2	Forearm	14
5	M	19	nodular	nd	1	Ear	48
6	F	21	nodular	10×10	1	Lower lip	6
7	M	23	nodular	10×10	1	Nose	6
8	M	16	nodular	20×23/10×5	2	Forehead	4
9	M	11	nodular	47×30	1	Forehead	12
10	M	21	mixed	nd	2	Cheek/ear	14
11	F	11	nodular	10×30	1	Cheek	12
12	M	19	nodular	10×25	1	Nose	36
13	M	20	nodular	40×80/nd	2	Neck	4
14	F	50	nodular	30×40	1	Nose	4
15	F	12	nodular	nd	1	finger	6

nd = not done.

### Arginase activity and phenotype of arginase-expressing cells in PBMCs

**Figure 1:**
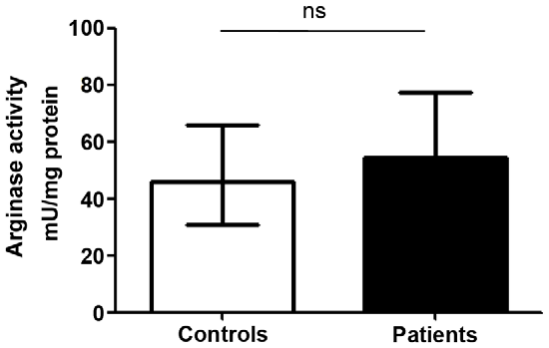
Arginase activity in PBMCs from controls and LCL patients. PBMCs from controls (n = 10) and LCL patients (n = 11) were isolated by Ficoll gradient and the activity of arginase was measured by enzymatic assay. Values represent median with interquartile range. Statistical significance was determined by a two-tailed Mann-Whitney test, ns = not significant.

**Figure 2:**
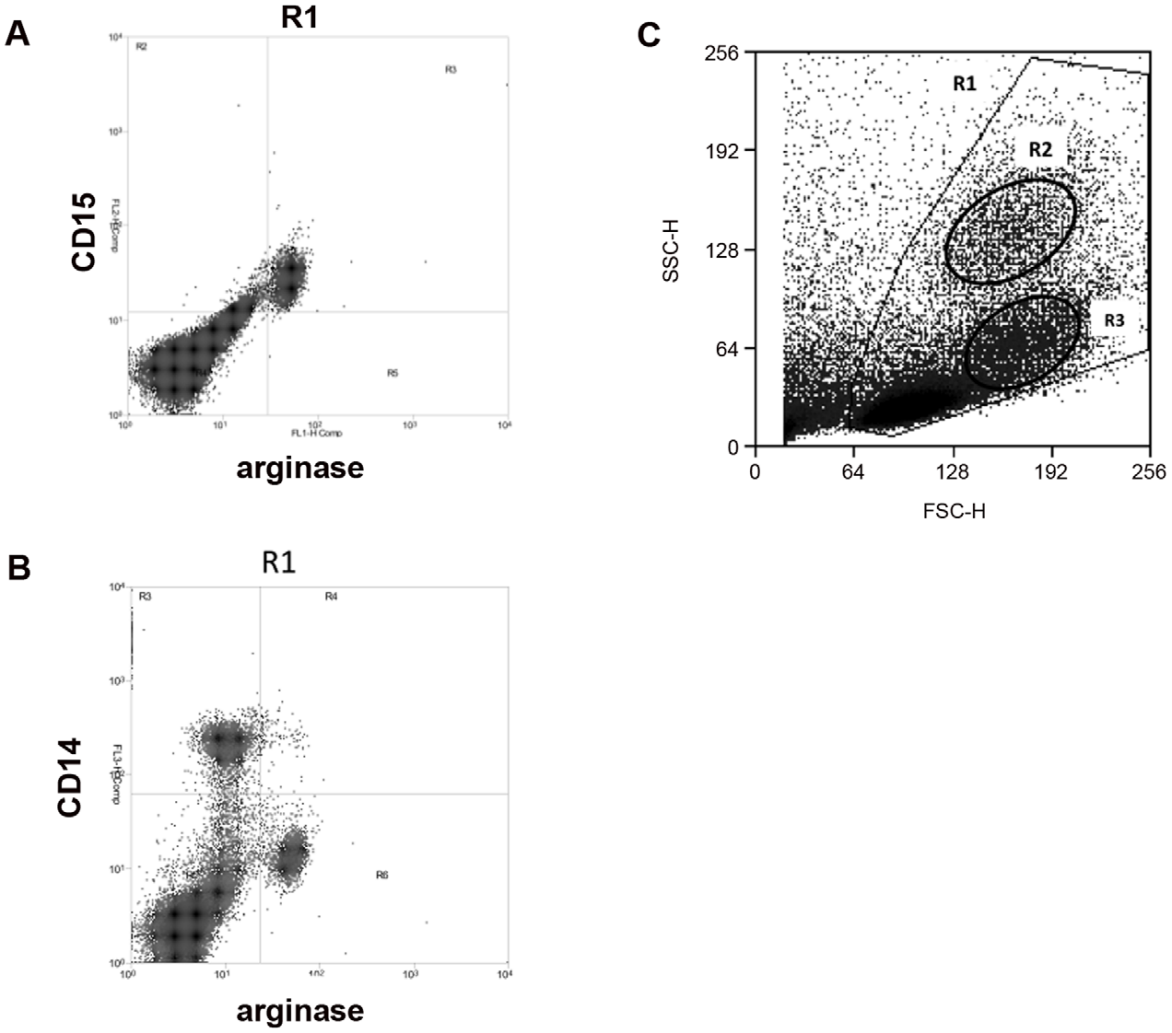
Arginase-expressing cells in PBMCs are neutrophils. PBMCs were isolated by density gradient from the blood of LCL patients. The phenotype of arginase-expressing cells was determined by flow cytometry. (A) Dot plot profile of CD15^+^ arginase^+^ cells; (B) dot plot profile of CD14^+^ arginase^+^ cells; (C) dot plot profile of forward (FSC) and side scatter (SSC). Data show the results of one representative experiment out of 15 independent experiments. Isotype control for arginase: 0.98%.

**Figure 3:**
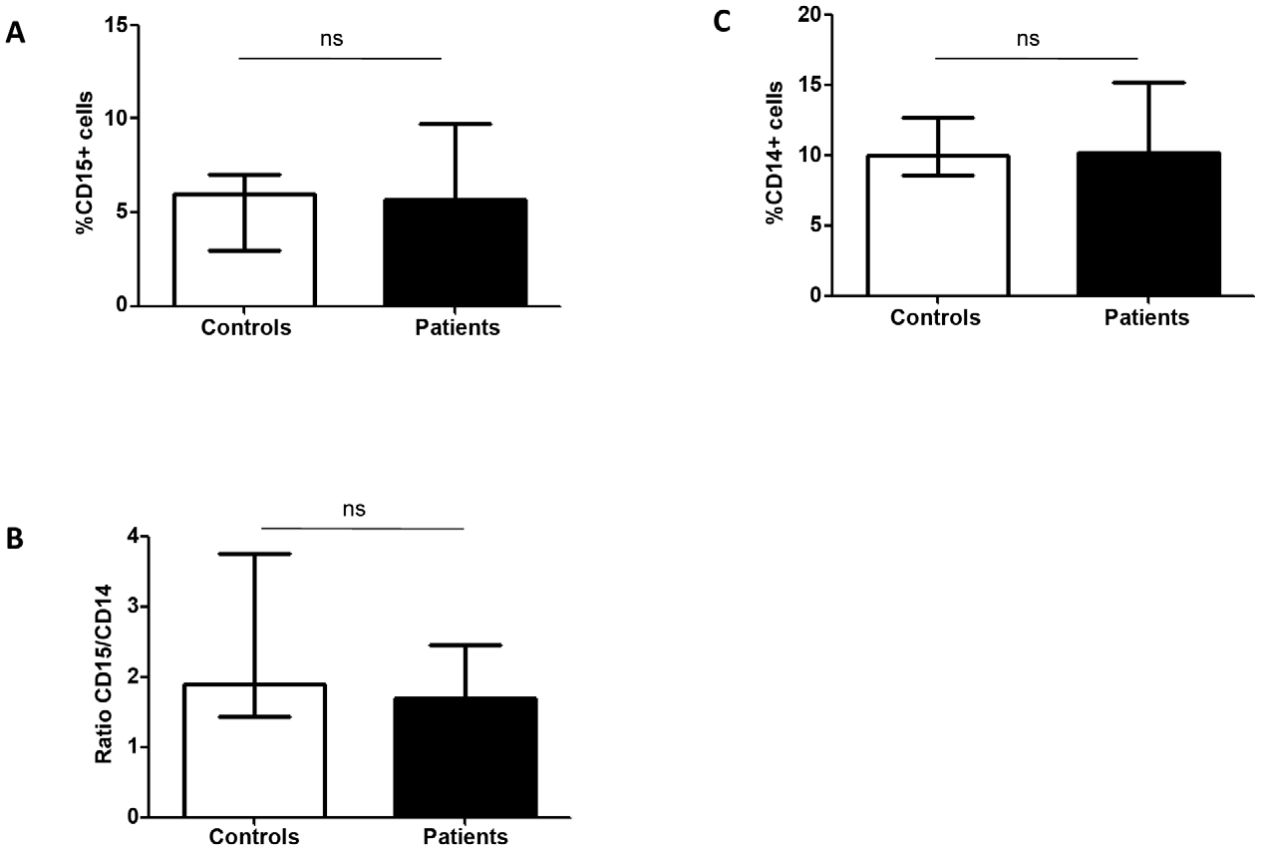
Frequency of neutrophils and monocytes in PBMCs. PBMCs were isolated by density gradient from the blood of controls (n = 10) and LCL patients (n = 15) and the frequencies of CD15^+^ (A), CD14^+^ (B) cells and the ratio of CD15/CD14 (C) were determined by flow cytometry. Values represent median with interquartile range. Statistical significance was determined by a two-tailed Mann-Whitney test, ns = not significant.

We first assessed the levels of arginase activity in PBMCs of LCL patients and compared it with healthy controls. The arginase activity was not statistically increased in the PBMCs of LCL patients (54.5 vs 45.1 mU/mg protein, *p* = 0.2751, Figure 1). We then determined the phenotype of arginase expressing cells in the PBMCs of LCL patients and controls and the results showed that the cells expressing arginase are low-density granulocytes (LDGs = CD15^+^ CD14^low^, Figure 2A). In all 15 patients tested, >93% of CD15^+^ cells expressed arginase. Similarly, the large majority of arginase-expressing cells in the PBMCs obtained from the controls were CD15^+^ (data not illustrated). In contrast, the frequency of arginase-expressing monocytes (CD14^+^ CD15^−^ arginase^+^) was below 1% (Figure 2B), except for 2 patients (1.1% and 1.2%, data not illustrated). Both cells types - LDGs and monocytes - were present in distinct regions of the forward and side scatter (FSC/SSC) dotplot: LDGs were found in region R2 (Figure 2C) and monocytes in region R3 (Figure 2C). The frequencies of LDGs (Figure 3A), monocytes (Figure 3B) and the ratio of LDGs/monocytes (Figure 3C) in PBMCs were similar between controls and LCL patients (median±sem: %CD15^+^: 6.0±0.66 vs 5.7±1.16; %CD14^+^ cells: 10.0±1.48 vs 10.2±1.21; ratio CD15^+^/CD14^+^: 1.90±0.43 vs 1.7±0.71, *p*>0.05).

The results presented in Figures 1, 2, and 3 show that the arginase activity and frequency of arginase-expressing cells in PBMCs of LCL patients are not significantly increased. These results also establish that the arginase-expressing cells in the blood of patients and controls are neutrophils.

### Arginase activity in lesions of LCL patients

**Figure 4:**
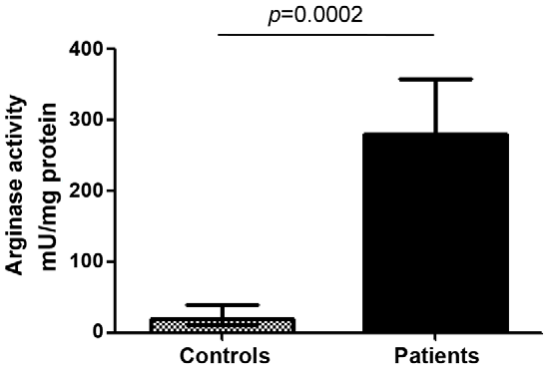
Arginase activity in skin lesions from controls and LCL patients. Skin biopsies from controls (n = 6) and LCL patients (n = 10) were homogenized and the activity of arginase was measured by enzymatic assay. Values represent median with interquartile range. Statistical significance was determined by a two-tailed Mann-Whitney test.

As shown in Figure 4, high levels of arginase activity were measured in lesions of LCL patients; notably, they were significantly higher than those measured in intact control skin (279.2±41.1 vs 18.8±6.3 mU/mg protein, *p* = 0.0002). There was no significant correlation between arginase activities and lesions size or duration of lesions (*p*>0.05).

**Figure 5:**
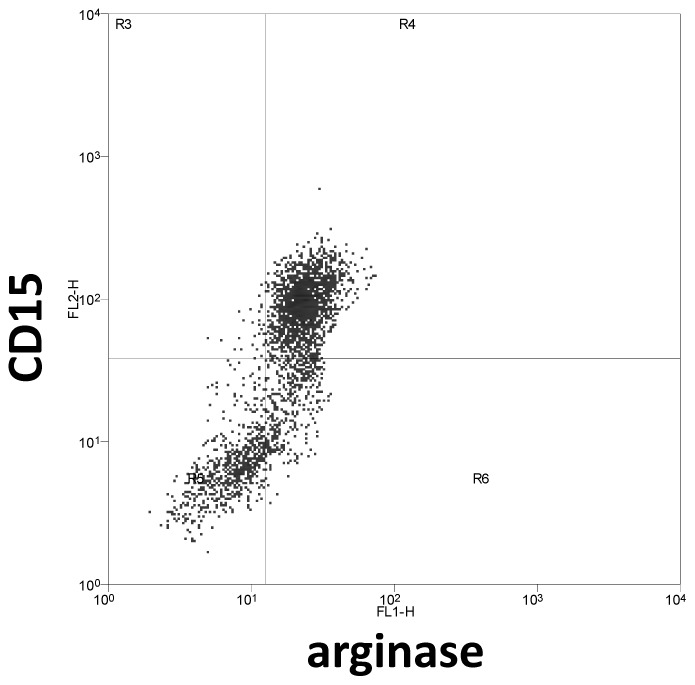
Arginase-expressing cells in biopsy are neutrophils. The phenotype of arginase-expressing cells in homogenates of skin biopsies was determined by flow cytometry by using a combination of antibodies against CD14, CD15 and arginase. Data show the results of one representative experiment out of three independent experiments.

To identify the phenotype of arginase-expressing cells in the lesions, we used the same combination of cell surface and intracellular arginase labeling as for the PBMCs. We were able to isolate enough cells (>1000 CD15^+^ cells) to be analyzed by flow cytometry from homogenates of skin biopsy from 3 out of 10 patients, who had a 4 mm biopsy taken. A CD15^+^ population was detected in patients' biopsy (Figure 5), in a region that was similar to that of LDGs detected in the PBMCs of the same patient (FSS/SSC: LDGs = 95/85, PBMCs = 101/82). In contrast, the frequency of CD14^+^ cells in the biopsies was very low (<250 events) and it was therefore not possible to characterise these cells in detail.

These results show that arginase activity is considerably increased in biopsied skin lesions of LCL in Ethiopia and suggest that arginase-expressing CD15^+^ cells are also present in the lesions of patients with cutaneous leishmaniasis in Ethiopia.

### Comparison of expression levels of CD3ζ, CD4 and CD8 in biopsies and PBMCs

**Figure 6:**
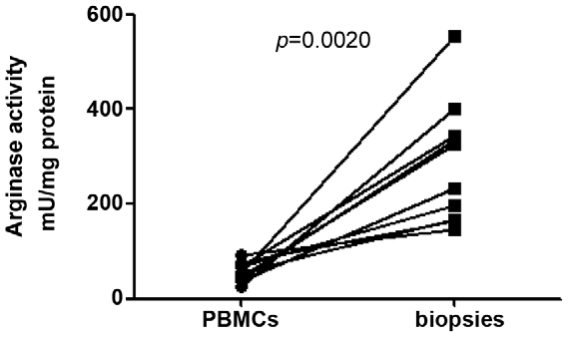
Arginase activity in cells isolated from peripheral blood and skin biopsies from LCL patients. Arginase activity was measured by enzymatic assay in skin homogenates and PBMCs from LCL patients (n = 10). Statistical significance was determined by a Wilcoxon paired test.

**Figure 7:**
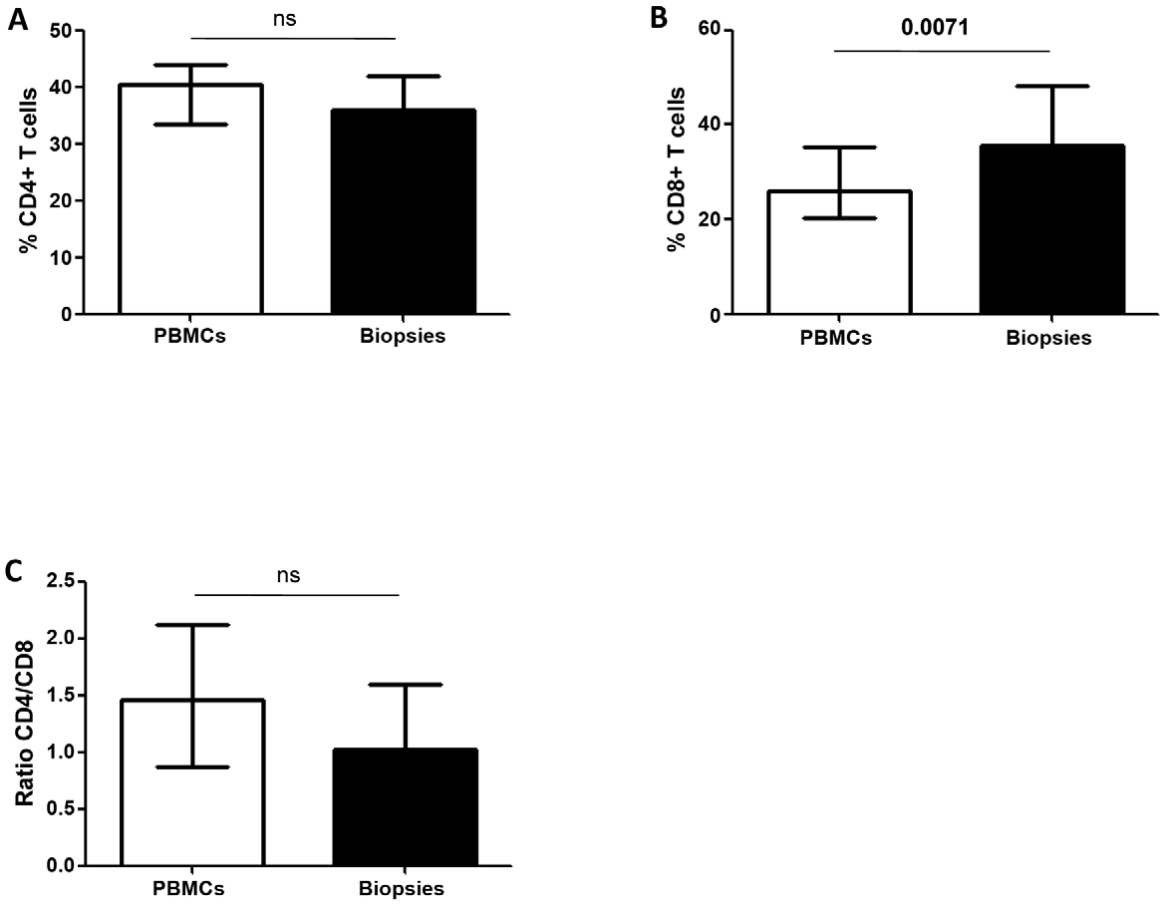
Frequency of CD4^+^ and CD8^+^ T cells in PBMCs and biopsies of LCL patients. The frequencies of CD4^+^ and CD8^+^ T cells was determined in cells isolated from the blood and from the biopsies of LCL patients (n = 15) by flow cytometry. (A) % of CD4^+^ T cells; (B) % of CD8^+^ T cells; (C) ratio of CD4/CD8. Values represent median with interquartile range. Statistical significance was determined by a two-tailed Mann-Whitney test, ns = not significant.

**Figure 8:**
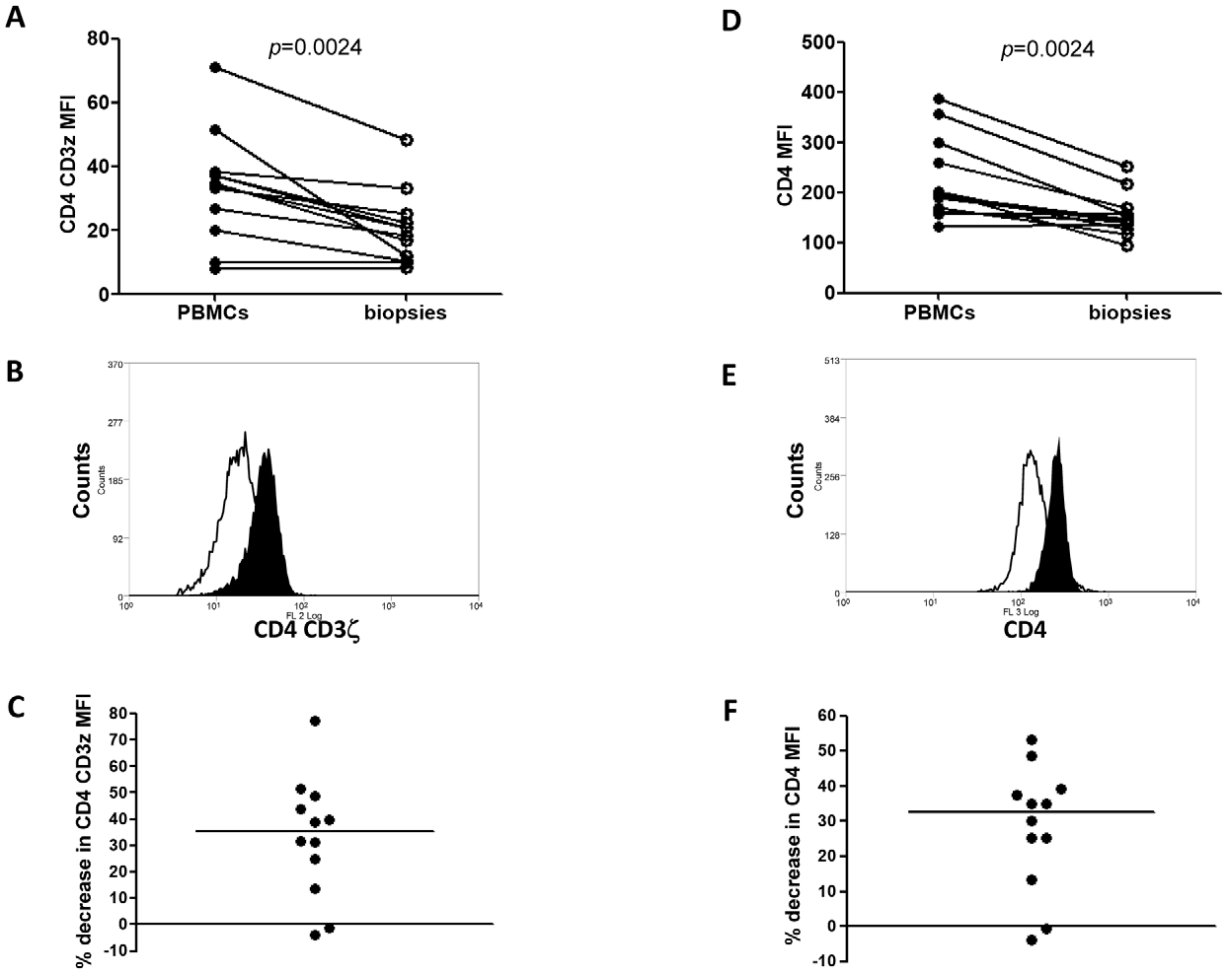
CD3ζ and CD4 MFI in PBMCs and biopsies of LCL patients. The MFI of CD3ζ in CD4^+^ T cells (A) and MFI of CD4 molecule (D) were compared between cells isolated from the blood and from the biopsies of LCL patients (n = 12) by flow cytometry; (B) representative histogram of MFI of CD3ζ in CD4^+^ T cells (open histogram  =  biopsies, closed histogram = PBMCs); (E) representative histogram of MFI of CD4 (open histogram = biopsies, closed histogram = PBMCs); (C) % decrease in CD3ζ MFI in CD4 (black bar = median); (F) % decrease in CD4. Statistical significance was determined by a Wilcoxon paired test.

**Figure 9:**
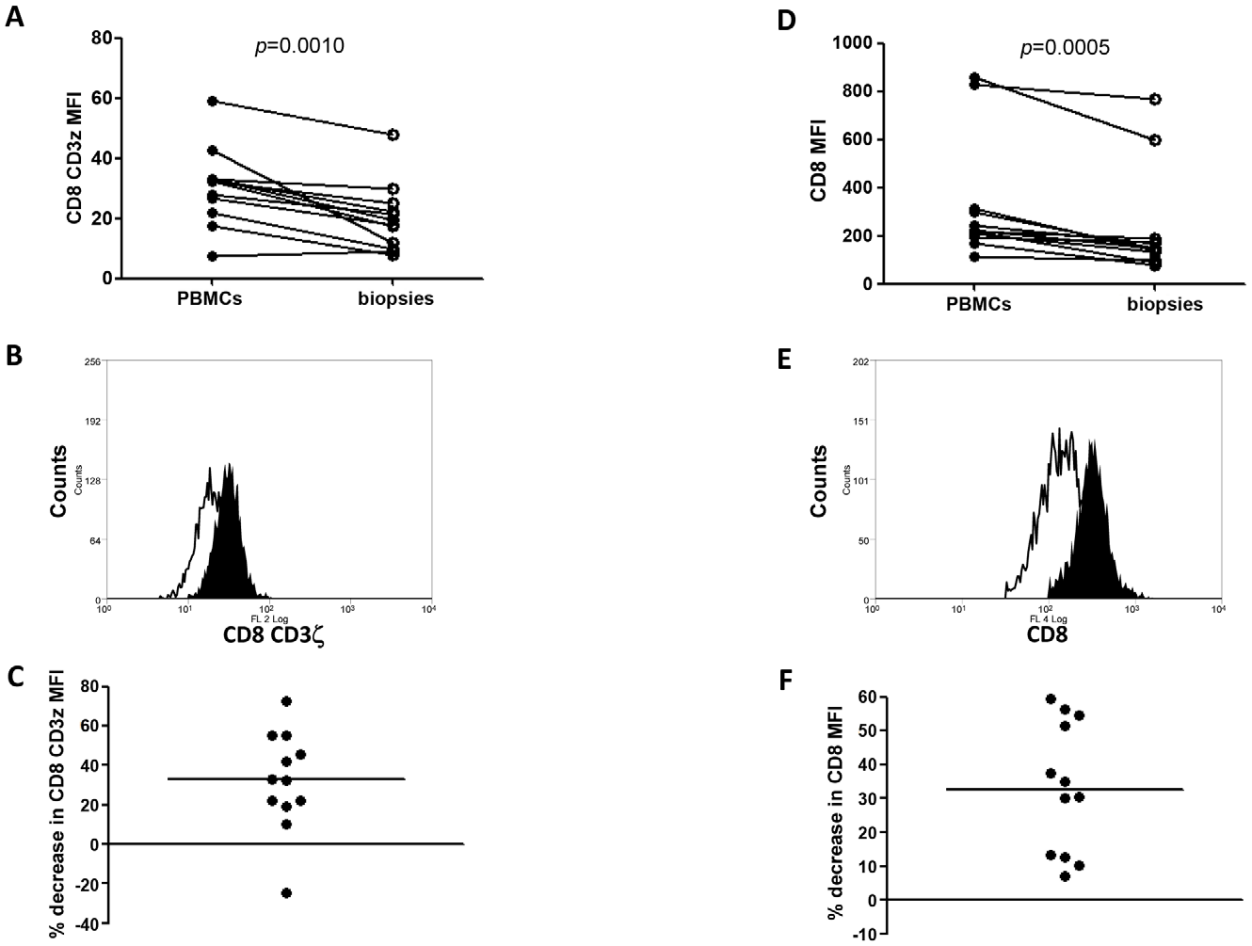
CD3ζ and CD8 MFI in PBMCs and biopsies of LCL patients. The MFI of CD3ζ in CD8^+^ T cells (A) and MFI of CD8 molecule (D) were compared between cells isolated from the blood and from the biopsies of LCL patients (n = 12) by flow cytometry; (B) representative histogram of MFI of CD3ζ in CD8^+^ T cells (open histogram = biopsies, closed histogram = PBMCs); (E) representative histogram of MFI of CD8 (open histogram = biopsies, closed histogram = PBMCs); (C) % decrease in CD3ζ MFI in CD8 (black bar = median); (F) % decrease in CD8. Statistical significance was determined by a Wilcoxon paired test.

Our results depicted in Figures 1 and 4 and summarized in Figure 6 show that cells isolated from cutaneous lesions express significantly higher levels of arginase activity per mg of protein as compared to cells isolated from peripheral blood of the same LCL patients (n = 10, 279.2±41.1 vs 53.4±6.4, *p* = 0.0020). We have previously shown that in a mouse model of CL, high arginase activity causes depletion of L-arginine, which impairs antigen-specific T cell responses [33]. Decreased expression of CD3ζ in T cells has been extensively used as marker of arginase-induced T cell suppression [24], [25]. Therefore, here we determined whether high arginase activity observed in the cutaneous lesions of patients coincides with lower expression levels of CD3ζ in CD4^+^ and CD8^+^ T cells as compared to those in the peripheral blood. First we compared the frequency and ratio of CD4^+^ and CD8^+^ T cells in the blood and the biopsies and show that the percentage of CD4^+^ T cells is similar in both compartments (Figure 7A). Interestingly, there was a higher frequency of CD8^+^ T cells in biopsies (*p* = 0.0071, Figure 7B). The ratio of CD4/CD8^+^ T cells was higher in the PBMCs, but it was not statistically significant (*p* = 0.075, Figure 7C). Of note, these frequencies and ratios were also comparable between PBMCs isolated from the blood from controls and patients (Table 2). Next, we measured the mean fluorescence intensity (MFI) of CD3ζ in T cells from homogenates of skin biopsy and compared it to those in cells isolated from the blood of the same patient. Results in Figures 8A–C show that the CD3ζ MFI in CD4^+^ T cells was lower in the biopsies of 10 out of 12 patients (*p* = 0.0024). Interestingly, the MFI of the CD4 molecule on T cells (CD4 MFI) was also lower in the biopsies as compared to the blood in 10 out of 12 patients (*p* = 0.0024, Figures 8D–F). Similar results were obtained with the expression levels of CD3ζ and CD8 molecules: it was decreased in 11 out of 12 patients tested (*p* = 0.0010, Figures 9A–C); moreover, CD8 MFI were lower in the biopsies of all patients tested (*p* = 0.0005, Figures 9D–F).

**Table 2:** Ratio of CD4/CD8^+^ T cells in PBMCs isolated from controls (n = 10) and LCL patients (n = 12).

	% CD4	% CD8	CD4/CD8
Controls	39.6+/−2.2	26.2+/−2.6	1.4+/−0.1
Patients	40.2+/−2.0	25.6+/−2.0	1.4+/−0.2

Values represent median ± sem.

These results show that the expression levels of CD3ζ, as well as CD4 and CD8 molecules are reduced in the skin biopsies as compared to those present in the blood of the same patient.

## Discussion

Here we showed that our results obtained in a mouse model translate to human disease: the levels of arginase activity were similar in the cells isolated from peripheral blood of LCL patients and controls, showing that arginase is not increased in the periphery following infection with *Leishmania* parasites. In sharp contrast, arginase was clearly increased in skin lesions of LCL patients as compared to intact skin. Moreover, arginase activity was considerably higher in the cells isolated from the skin biopsies as compared to the PBMCs. These results are in agreement with those obtained in the mouse model, where we showed that arginase is upregulated at the site of pathology, but not in the periphery [33].

The cells expressing arginase were identified as neutrophils both in the blood and in the lesions. In the blood, these cells co-purify with PBMCs and have therefore been named low-density granulocytes (LDGs). We and others have already described these cells [36] [28], [35], [37] and have shown that they were likely to be activated granulocytes [38]. Whereas we have previously shown that bone marrow derived macrophages activated with IL-4 or IL-4 and IL-10 upregulate arginase [32], [39], [40], we have not yet identified the phenotype of arginase-expressing cells in lesions of BALB/c mice infected with *L. major*. We have previously shown that both macrophages and neutrophils are recruited into lesions of *L. major* infected mice [41], [42], therefore we cannot exclude that in these lesions, macrophage and/or neutrophils express arginase. We could identify only low numbers of macrophages in the lesions (<250 events), this was unexpected as *Leishmania*-infected macrophages can be identified in scrapings from cutaneous lesions and in fixed biopsies [43], [44]. It is possible that the technique used to homogenize the lesions damages the macrophages and it is therefore not possible to conclude from this study whether macrophages also contribute to the overall arginase measured in the lesions.

Increased arginase activity in macrophages has been shown to favour parasite growth [32], [45]. *Leishmania* parasites are taken up by neutrophils (reviewed in [46], [47] and it is possible that this gives them a survival advantage. However, since they have not been shown proliferate efficiently in neutrophils, here we favour the hypothesis that neutrophil arginase affects both wound healing (1) and T cell responses (2), two processes that are crucial in resolving cutaneous lesions:

1. L-arginine is the substrate for both arginase and inducible nitric oxide synthase (iNOS), which play an important role in wound healing. Increased synthesis of nitric oxide (NO) by NOS promotes processes such as angiogenesis, epithelialisation and collagen synthesis, which are critical for tissue repair [48], [49], [50]. On the other hand, arginase catabolises L-arginine into ornithine, an essential precursor of polyamines and proline, which are both required for wound healing. Therefore, it is possible that arginase is increased in lesions of LCL patients as part of the normal process of tissue repair. However, arginase has also been shown to be overexpressed in pathological wounds such as those found in psoriasis [51], [52] and diabetes [53]. Since NO production is reduced by arginase via the depletion of L-arginine [54], it is possible that upregulation of arginase might reduce the production of NO, thereby not only compromising tissue repair, but also parasite killing. Further, a study by Kavalukas et al. has recently shown that arginase activity can delay wound healing [55], thus arginase may have positive as well as detrimental roles depending on the duration and the type of lesions.
2. Arginase-induced L-arginine catabolism is a well-established mechanism of immune suppression [24], [56] [26]. Depletion of L-arginine has been shown in superficial wounds [50], in psoriatic lesions [51] and lesion from *L. major*-infected mice [33]. To assess whether high arginase expressed in the lesions of LCL patients coincides with dysfunctional T cell responses we measured the expression levels of CD3ζ in T cells. CD3ζ is widely used as a marker of impaired T cell activation: its downregulation coincides with impaired T cell functions such as proliferation and cytokine production [57], [58], [59]. The results presented here show that the MFI of CD3ζ is lower in the CD4^+^ and CD8^+^ T cells isolated from the skin biopsies as compared to those from the blood, suggesting that T cell activation and function might be compromised in lesions from LCL patients; this might contribute to their inability to mount efficient immune responses to control parasite load and resolve the lesions. Further, our results also show that the expression levels of CD4 and CD8 molecules are lower on T cells isolated from the biopsies as compared to those isolated from peripheral blood. Antigenic modulation of the expression levels of these molecules has already been shown [60], [61]. Taken together, our results suggest that upregulation of arginase in lesions of LCL patients coincides with functional impairment of T cells.

Persistent leishmaniasis has been associated with immune suppression [20], [21], [22]. The biopsies analyzed in the present study were all collected from LCL patients. The observed natural history of LCL is that ∼70% heal within 12 months and 30% within 24 months [7]. However, it is not possible to predict whether a lesion will heal or become chronic and indeed, 8 patients had their lesions for 12 months or more. Therefore, we propose that high arginase results in impaired T cell responses and therefore contributes to the delay of healing that is characteristic in LCL in Ethiopia. Of note, the majority of LCL lesions analysed here were nodular and we cannot exclude that ulcerated lesions might express different levels of arginase and/or CD3ζ. The results of this study call for further work to analyse in detail different types of LCL lesions.

Cutaneous leishmaniasis causes serious morbidity in Ethiopia. The lesions, which are most commonly on the face, are chronic, disfiguring and sometimes disabling, and cause significant social stigma [62]. To date, the immunopathology of this disease has been poorly understood. Here we show for the first time that arginase is upregulated in lesions of patients with LCL and that this coincides with reduced levels of CD3ζ expression in T cells. Further study is needed to assess whether arginase-mediated L-arginine metabolism is a key element in the outcome of human leishmaniasis and this is currently ongoing in our laboratories. Therapeutic interventions that can regulate arginase and L-arginine metabolism might prove useful in the treatment of cutaneous leishmaniasis, and possibly in visceral leishmaniasis.
